# Exploring a Clinically Friendly Web-Based Approach to Clinical Decision Support Linked to the Electronic Health Record: Design Philosophy, Prototype Implementation, and Framework for Assessment

**DOI:** 10.2196/medinform.3586

**Published:** 2014-08-18

**Authors:** Perry Miller, Michael Phipps, Sharmila Chatterjee, Nallakkandi Rajeevan, Forrest Levin, Sandra Frawley, Hajime Tokuno

**Affiliations:** ^1^VA Connecticut Healthcare SystemWest Haven, CTUnited States; ^2^Center for Medical InformaticsYale University School of MedicineNew Haven, CTUnited States; ^3^Department of AnesthesiologyYale University School of MedicineNew Haven, CTUnited States; ^4^Baltimore VA Medical CenterBaltimore, MDUnited States; ^5^Department of NeurologyUniversity of Maryland School of MedicineBaltimore, MDUnited States; ^6^Department of MedicineYale University School of MedicineNew Haven, CTUnited States; ^7^Department of NeurologyYale University School of MedicineNew Haven, CTUnited States

**Keywords:** Internet, clinical decision support systems, electronic health records, neuropathic pain, therapeutics

## Abstract

**Background:**

Computer-based clinical decision support (CDS) is an important component of the electronic health record (EHR). As an increasing amount of CDS is implemented, it will be important that this be accomplished in a fashion that assists in clinical decision making without imposing unacceptable demands and burdens upon the provider’s practice.

**Objective:**

The objective of our study was to explore an approach that allows CDS to be clinician-friendly from a variety of perspectives, to build a prototype implementation that illustrates features of the approach, and to gain experience with a pilot framework for assessment.

**Methods:**

The paper first discusses the project’s design philosophy and goals. It then describes a prototype implementation (Neuropath/CDS) that explores the approach in the domain of neuropathic pain and in the context of the US Veterans Administration EHR. Finally, the paper discusses a framework for assessing the approach, illustrated by a pilot assessment of Neuropath/CDS.

**Results:**

The paper describes the operation and technical design of Neuropath/CDS, as well as the results of the pilot assessment, which emphasize the four areas of focus, scope, content, and presentation.

**Conclusions:**

The work to date has allowed us to explore various design and implementation issues relating to the approach illustrated in Neuropath/CDS, as well as the development and pilot application of a framework for assessment.

## Introduction

This paper describes a project that is exploring an approach to computer-based clinical decision support (CDS), built using Web technologies and linked to the electronic health record (EHR). The goal of the project is to explore ways in which CDS can be made clinician-friendly from a variety of perspectives. We believe that this is an important goal that needs to be addressed explicitly. This project is one step in that direction. The paper first discusses the project’s design philosophy. It then describes a prototype implementation (Neuropath/CDS) that explores the approach in the domain of neuropathic pain, in the context of the US Department of Veterans Affairs (VA) EHR. Finally the paper discusses a framework for assessing the approach, illustrated by a pilot assessment of the prototype.

Computer-based CDS is common in health care systems, especially those that have an EHR [1]. CDS applications serve a variety of purposes, including providing alerts and reminders to clinicians at the point of care and making patient-specific recommendations about treatment and medications. Other approaches to computer-based CDS include computer-based clinical practice guidelines [2], clinical "dashboards" that provide focused information about specific clinical issues [3], and clinical "info buttons" [4] designed to facilitate access to online information relevant to a patient's clinical status.

The VA's national EHR (which includes the Veterans Health Information Systems and Technology Architecture backend and the Computerized Patient Record System (CPRS) interface) has evolved over the course of several decades as a powerful tool for patient care. There is widespread agreement that the CPRS can be made more powerful by incorporating increasing amounts of CDS. It will be important that this be accomplished in a fashion that assists the provider in clinical decision making without imposing additional demands and burdens upon the provider’s practice. The present project complements research that is underway at other VA locations including the ATHENA system that provides decision support for hypertension [5], and for opioid therapy for chronic, noncancer pain [6,7].

Studies of CDS deployment have documented the importance of fitting CDS into the clinician’s workflow, and of providing information at the time and place of clinical decision making [8]. The present work explores one approach to addressing these issues and a number of challenges that must be confronted, with a particular focus on accomplishing the goal in a clinically friendly fashion.

## Methods

### Design Philosophy

The overall goal of the project is to develop and explore an approach to computer-based CDS that is clinically efficient and clinician friendly. We want to include information that will be particularly useful to help the clinician manage a particular patient and make it easily accessible.

- The system should focus on a well defined, constrained clinical domain whose scope is easily understood by a clinician. Particularly important in this regard are the issues of focus and scope discussed below in the section describing our pilot framework for assessment.

- The system should be easy to use. For example: (1) it should be easy to invoke from the EHR, (2) it should be able to extract clinical data from the EHR rapidly, (3) the clinician should be able to inspect the information provided rapidly and easily, and (4) use of the system should be optional, not required.

- The system should provide a range of potentially useful information, accessible “in one place”.

- The system should present material in a flexible intuitive fashion.

- The system should provide information to help the clinician decide how to manage the patient, but not try to tell the clinician what to do next.

### A Prototype Implementation- Neuropath/CDS

Neuropath/CDS is a prototype CDS system developed to explore the issues involved in bringing computer-based CDS to the clinician at the point of care in a fashion that fits efficiently into the busy clinical environment. Neuropath/CDS uses patient data automatically extracted from the VA EHR to assist the primary care provider (PCP) in decisions regarding the first-line pharmacologic management of neuropathic pain (NP). NP is a chronic neurological condition that often requires continual monitoring and adjustment of treatment [9-11]. Ongoing clinical trials have generated repeated revisions of guidelines [9,12]. Multiple attempts at treatment with different drugs or drug combinations are often necessary, with drug effectiveness and side effects varying among different patients.

Neuropath/CDS has been refined in collaboration with the Neurology Service at the VA Connecticut Healthcare System (VACHS). Key features of the approach include the following.

- The system is an optional adjunct to care, accessible via a drop-down "Tools" menu from the EHR interface.

- It is driven from the patient record, retrieving patient demographics, comorbidity information, as well as current and past drug prescription information in a few seconds.

- The information provided is accessible from a single screen that we expect can be typically processed by the clinician user in well under a minute.

- Comments and recommendations are not prescriptive (ie, do not tell the clinician how to manage the patient), but rather describe options for management tailored to the patient's comorbidities and current treatment regimen.

- A visual outline of pharmacologic management is also provided, with “hover boxes” that provide further information about pharmacologic options. Several links to additional information are also provided.

In this section, we first show an example of Neuropath/CDS in operation and describe the various components of its clinical user interface. We then describe the system's technical design, and our pilot framework for assessment.

### Neuropath/CDS in Operation

#### Clinical Interface

**Figure 1:**
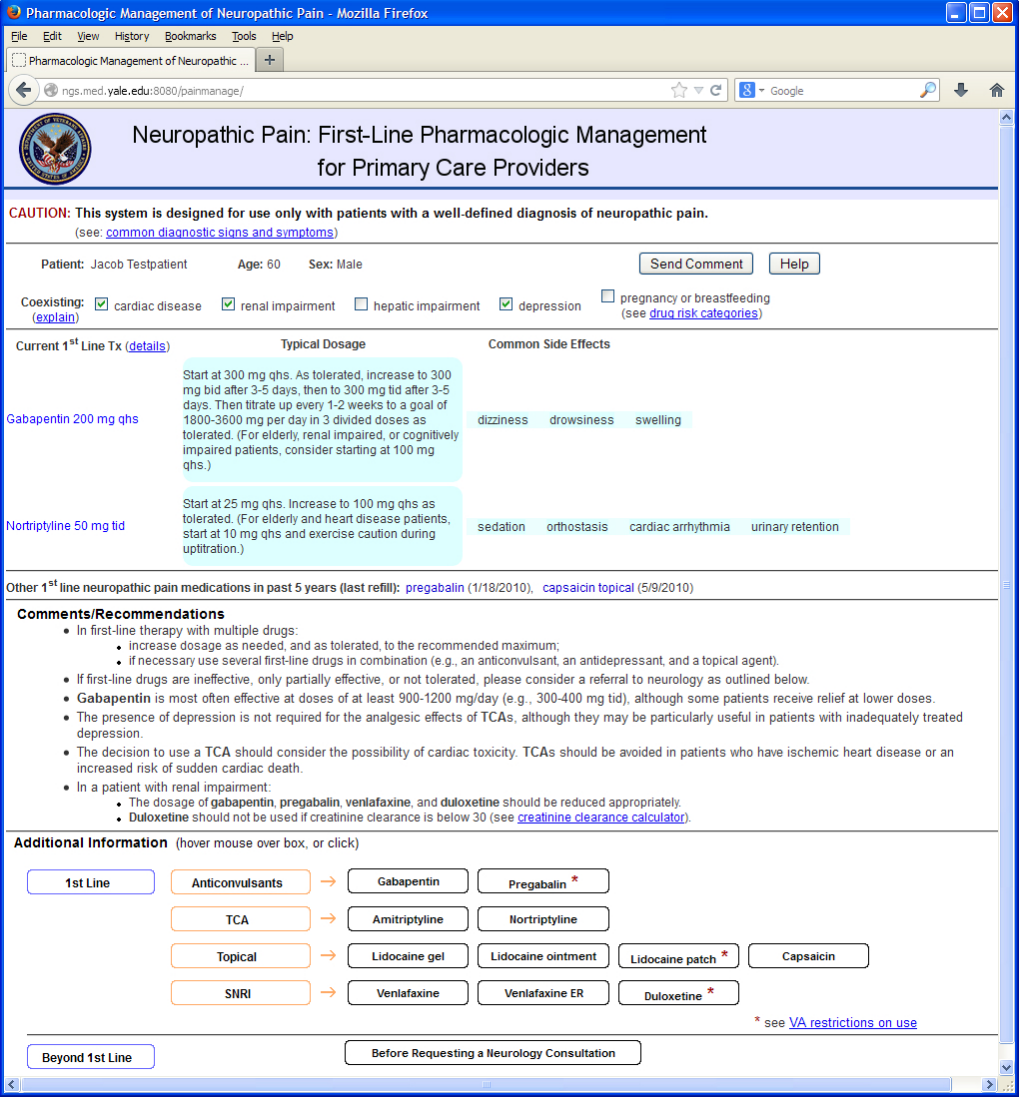
The Web-based clinical interface of Neuropath/CDS, as described in the text. TCA=tricyclic antidepressant, SNRI=serotonin–norepinephrine reuptake inhibitor.

Figure 1 shows the Web-based clinical interface of Neuropath/CDS. To view this interface, the clinician must select a patient within CPRS and invoke Neuropath/CDS using the "Tools" menu located at the top of the CPRS screen. The system typically takes 5-10 seconds to gather data from the EHR and present this interface. Since the system is still under development, a password is currently required. The clinical interface includes the following components.

#### Basic Patient Data

Near the top of the screen are the patient’s name (in this example, a test patient), age, sex, and a set of check boxes indicating the presence or absence of comorbid disease relevant to NP. These checkboxes are set automatically based on International Classification of Diseases, 9th Revision (ICD9) codes in the EHR, but can be changed (checked or unchecked) by the provider based on his/her knowledge of the patient's clinical status.

#### Current and Past Neuropathic Pain Medications

A little lower on the screen is information about the patient’s current NP medications, which is followed by information about any other NP medications taken during the past five years, all extracted automatically from the EHR. For the patient's current first-line NP medications, information about dosage and common side effects is also shown.

#### Comments and Recommendations

The next section shows a set of “Comments/Recommendations”. These items are generated by if-then logic, and are tailored to the patient’s age, sex, comorbid diseases, and current NP medications. The goal is not to try to tell the clinician what to do next, but rather to present relevant patient-specific issues to be considered in making such a decision.

#### A Visual "Hover Box" Outline of the Domain

**Figure 2:**
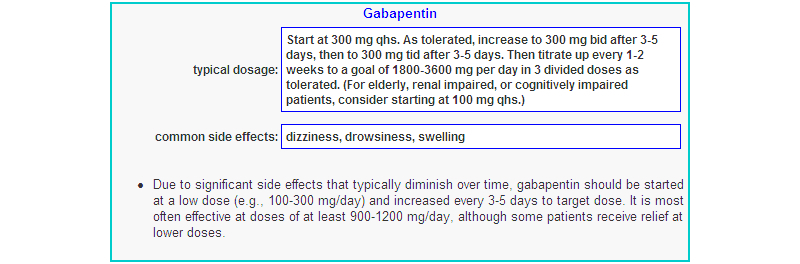
An example "hover box" that appears when the user "hovers" the mouse over the box labeled "Gabapentin" near the bottom of the screen shown in Figure 1.

**Figure 3:**
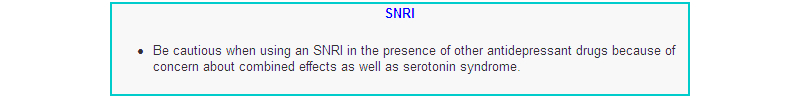
An example "hover box" linked to the box labeled "SNRI". SNRI=serotonin–norepinephrine reuptake inhibitor.

**Figure 4:**
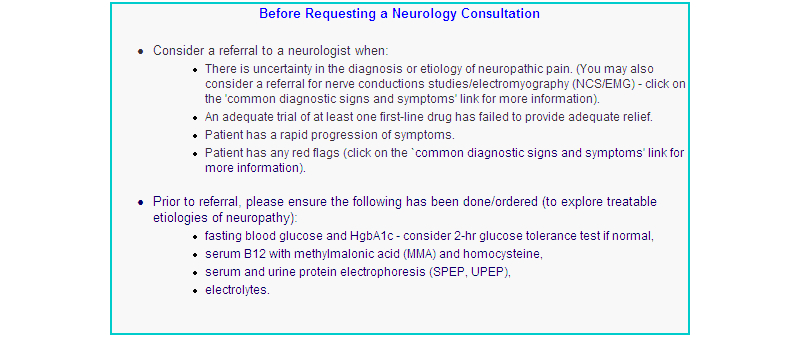
An example "hover box" linked to the box labeled "Before Requesting a Neurology Consultation".

The bottom of the screen presents a visual outline of first-line pharmacologic management of NP. If the user moves the computer mouse over one of the item names, a temporary “hover box” appears on the screen containing information about that item. Figures 2-4 show these boxes. The goal is to allow the clinician to explore alternatives for the patient's first-line NP management. The information presented can be conditionally tailored to the patient by if-then logic. If the user clicks on one of the item names, this information is presented in a pop-up box, which can be saved, printed, or copied into the patient record.

#### Links to Static Web Pages Providing Focused Clinical Information

The interface also provides a limited number of links to static Web pages that provide additional information, for instance: (1) to assist in making the diagnosis of NP, (2) to provide pregnancy and lactation risk information for NP medications, and (3) to provide instructions regarding the steps required before using drugs that are not part of the normal VA formulary.

#### Overview of Neuropath/CDS’s Technical Design

**Figure 5:**
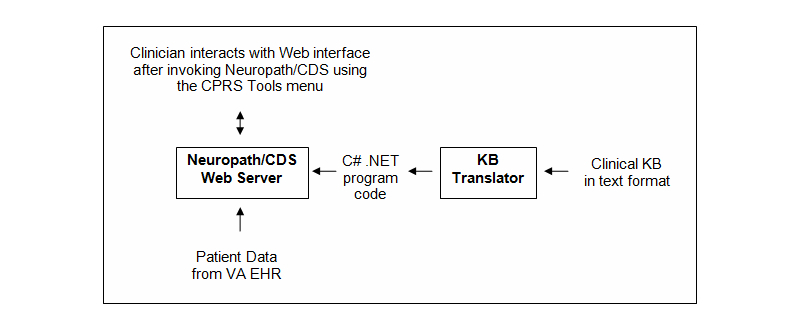
A simplified schematic outline of Neuropath/CDS's technical design. KB=knowledge base; CPRS=computerized patient record system; VA=Veterans Administration; EHR=electronic health record.

Figure 5 shows a simplified schematic overview of Neuropath/CDS's technical design. When the clinician invokes Neuropath/CDS from CPRS using the Tools menu, a Web server program is activated with the patient’s numeric identifier (ID) passed as a parameter. The Web server then retrieves patient data from the EHR as described below, and uses it to fill in the clinical values in the Web interface.

#### Automatic Extraction of Patient Data From the Electronic Health Record

The key factor in allowing Neuropath/CDS to operate with clinical data retrieved from the EHR is that this must be done quickly, in a clinically acceptable timeframe. Performing straightforward database calls to the EHR (which contains millions of rows of data not optimized for direct querying) would take much too long. We have taken two approaches to deal with this problem.

A variety of Web service procedures have been developed within VA EHR to facilitate the very rapid retrieval of selected patient-specific data. Neuropath/CDS uses these to retrieve the patient’s current medications, from which the current NP medications are extracted.

For certain data (ICD9 codes and previous NP medications), a program runs through the entire VACHS patient database during the night to extract and condense the appropriate patient data into a much smaller "data staging" database that can be rapidly queried. For example, based on an examination of ICD9 codes in the EHR, a bit is set in the staging database reflecting the presence or absence of heart disease for each patient. Neuropath/CDS queries this smaller database to retrieve patient-specific data on comorbid disease and previous NP medications.

#### Clinical Knowledge Base

The Neuropath/CDS knowledge base (KB) consists of if-then logic of two types: (1) if-then rules, and (2) conditional comments.

The simple if-then rule shown in Textbox 1 states, “if the patient is receiving amitriptyline treatment or nortriptyline treatment, then the patient is receiving TCA (tricyclic antidepressant) treatment". Before the KB logic is executed, the variables amitriptyline_tx and nortriptyline_tx are set appropriately to true or false (by program code) based on a patient’s clinical data.

The conditional comment shown in Textbox 2 states that: (1) "if the patient is receiving TCA treatment and has a history of depression", then a specified text comment should be included in the “Comments/Recommendations” section of the screen, and (2) "if the patient has a history of depression", then the text comment should also be included in the “TCA" hover box. In this way, text comments are directed to the different sections of the interface, where they are then sorted based on the “order” number (shown below), and displayed in sorted sequence.

Notice that this design allows a comment to be presented in different locations within the Web interface (eg, in the “Comments/Recommendations” section and/or in a “hover box”). A single comment can be directed to more than one screen location based on different if-then logic for each location. Comments can also be sorted and displayed in a different order in different locations.

The KB Translator automatically converts the KB into C# .NET code, which is then copied into a larger Web server program (built using Asynchronous JavaScript and Extensible Markup Language [AJAX], C# .NET, and cascading stylesheets) that implements Neuropath/CDS as a whole. By allowing the logic to be translated automatically into C# .NET code that is then run directly on the Web server (compared, for example, to using an interpretive rules engine package or some other complex knowledge manipulation environment), we achieve logic that runs very rapidly. Using this approach, the Web interface is dynamic. For example, if the user checks or unchecks one the checkboxes indicating the presence/absence of comorbid disease, the KB logic is immediately rerun and the system’s comments/recommendations on the Web screen are changed appropriately in a fraction of a second.

**A simple if-then rule.**

if (amitriptyline_tx

     || nortriptyline_tx)        tca_tx = true;

**A conditional comment.**

Comment TCA_depression {

Condition: tca_tx & depression_hx; Where: Recommendations (order: 7);

Condition: depression_hx; Where: TCA_comments (order: 5);

Text:          "The presence of depression is not required for the analgesic effects

                   of TCAs, although they may be particularly useful in patients with

                   inadequately treated depression." }

### Developing a Framework for Assessment- Scope, Focus, Content, and Presentation

To provide a structure for our analysis, we developed a framework structured around the four areas of focus, scope, content, and presentation, as described below. This framework may also prove useful to CDS researchers or developers who might wish to adopt this general approach to CDS or certain of its features. Although the dividing line between these four areas is not always distinct, we believe that they provide a framework that is useful for discussing our experience and the lessons learned to date, and potentially useful to others in the future.

The current implementation of Neuropath/CDS is the result of several years of iterative development that has involved multiple interactions with many VA clinicians and technical staff. This process started as a collaboration with the VACHS Pain Management Clinic. As the project matured, the primary focus of the clinical collaboration shifted to the VACHS Neurology Service, and its relationship to VACHS PCPs, as described in more detail below.

In addition to extensive collaboration with individual clinicians, the system was presented at several conferences within the VACHS and elsewhere. These sessions resulted in further refinement of the system's design. We then conducted a formative study, with IRB approval, involving eight structured sessions in which project staff sat with a VACHS clinician as he/she used the operational CPRS interface with real patients and some test patients. During these sessions, the clinicians interacted directly with the computer using the mouse and keyboard, with project staff verbally providing a discussion of the features and answers to questions. The clinicians were asked to "think aloud" as they interacted with the various features of the system, vocalizing whatever thoughts they were having, which included reactions, comments, suggestions, and questions. At the end of the session, the clinician was asked to talk more broadly about the system, its features, its potential utility, as well as suggestions of additional features, content, and functionality.

We were interested in clinician reactions to the current Neuropath/CDS system, as well as to the overall approach to CDS. Although clinicians were very positive about the system and approach, we did not focus on trying to quantitate this aspect of their assessment, since they knew they were speaking directly to members of the system development staff, and evaluative comments would therefore be potentially biased. Instead, we focused on the other aspects of their comments.

## Results

### Developing and Refining the Approach

Since this paper describes a research project exploring an approach to providing CDS in a fashion that fits naturally into the clinical environment, the methodology described above is a major part of the “results” of the project. In addition, in this section we discuss the results of applying the pilot framework for the assessment described above.

### Focus

The key factor that has shaped the refinement of Neuropath/CDS to its current design was the decision to focus the system's role on the clinical interface between the VACHS Neurology Service and VACHS PCPs. PCPs treat NP themselves, but when they need assistance they typically consult Neurology. Neuropath/CDS is therefore designed to help the PCP manage NP in the manner that Neurology would recommend. The system also suggests situations in which the PCP might decide to consult Neurology, and a recommended workup for the PCP to perform when requesting such a consult. Once defined, this focus on the interface between Neurology and PCPs provided a guiding context for all other aspects of the system's design.

### Specific Comments on Focus

None of our VACHS clinician subjects commented explicitly on this focused role for the system. They appeared to find it natural and logical in the VACHS clinical environment.

### Scope

Once this focus was defined, it became logical that the scope of the system's clinical domain should center on the first-line pharmacologic management of NP. This scope represents a markedly reduced subset of the possible clinical issues involving NP that we might have included. For example it does not include: (1) the diagnosis of NP, (2) second-line and third-line pharmacologic management of NP which involve the use of opioids and tramadol (second line) and drugs like carbamazepine, lamotrigine, topiramate, valproic acid, bupropion, citalopram, or paroxetine (third line), or (3) interventional nonpharmacologic approaches such as spinal cord stimulation, intravenous infusions, epidural injections, and nerve blocks.

The major reason for this restriction in scope was that the VACHS neurologists wanted to be consulted before PCPs went beyond first-line NP drugs. This also reflected a national VA goal to avoid the use of opioids as much as possible, and to have PCPs get as much utility out of first-line drugs as possible.

### Specific Comments on Scope

An unexpected finding when clinicians used the system with real patients was that there was potential ambiguity regarding the borderline between first-line NP treatment and more advanced NP treatment, especially if one just looks at the drugs the patient is receiving. Some patients receiving first-line treatment for NP were also at the same time receiving an opioid (a second-line NP drug) for some other pain problem (not NP), or a high potency antidepressant (for clinically severe depression) that could also be used as a third-line NP drug. This was a potential source of confusion for the clinician in understanding the system's role and interpreting its advice for such patients.

To attempt to clarify these issues, we added additional explanatory text to the interface, adjusted the way in which the different NP medications were presented, and added the following comment to be used with patients who were receiving second- and third-line NP drugs.

This system is designed ONLY for first-line pharmacologic management of neuropathic pain (NP). Some patients receive second- or third-line NP drugs for other reasons (not for NP), in which case the comments below may still be relevant. If this patient is receiving a second-line drug (eg, an opioid or tramadol) or a third-line drug (eg, carbamazepine, lamotrigine, topiramate, valproic acid, bupropion, citalopram, or paroxetine) for NP, and you have concerns about NP management, please consider referral to neurology.

Neuropath/CDS explanatory text

As we gain more experience with the use of Neuropath/CDS in the clinical environment, we may need to make further refinements to deal with this particular issue involving scope.

An additional issue related to scope, which generated considerable discussion during the system's development, involved how to deal with the diagnosis of NP. The concern was how best to make sure that the system was used for appropriate patients (ie, patients who indeed had NP). An approach might have been to include what would essentially be a second complementary interactive CDS system focused on NP diagnosis. It was decided that this was not necessary and that this issue could be addressed by including a link to a static Web page that outlined in tabular form the clinical signs and symptoms that could serve to confirm the diagnosis of NP, or to suggest other diagnoses. All of our subject clinicians read this page quite carefully, and several explicitly stated that the content was very good. They did not appear to believe that a different approach was needed, and none expressed any concern about this approach to diagnosis of NP.

### Content

Our main goal related to content was to provide useful practical information, relevant to immediate patient management. During the development of Neuropath/CDS, issues raised included the following.

A number of clinicians indicated that detailed dosage information would be very useful.

There was wide agreement that providing a list of first-line NP drugs that had been prescribed in the past would be particularly useful, since it could take up to 30-60 minutes of frustrating search through the EHR to find this information.

A succinct description of the steps required before a PCP could use a "nonformulary" drug was also identified as valuable. The VA document provided for each such drug is typically 6-8 pages long. The clinically relevant information could be condensed to a paragraph or two expressed as a bulleted outline.

A readily available outline of the pregnancy and lactation risk levels for the various NP drugs was also identified as useful to have available.

Many features such as these were added incrementally based on provider feedback as we developed and refined the system over time. Another major content-related goal was that the comments and recommendations made should not be prescriptive. The goal is to present relevant issues to be considered in making a decision in the context of a particular patient's current clinical status.

### Specific Comments on Content

Different clinicians raised a number of specific issues. For example: (1) Might the system provide more assistance in choosing among first-line drugs? (2) Might the system more explicitly address the desirability of not using opioids prematurely? (3) Should the system be more explicit about the need to use lower doses of drugs in the elderly? (4) Is it really necessary to order serum and urine protein electrophoresis (tests to rule out multiple myeloma) when requesting a Neurology consult? And (5) should the system recommend against the use of amitriptyline in a patient over 65 (a suggestion made by a geriatrician based on geriatric guidelines)?

The answers to questions of this sort are not always clear-cut. For example: (1) there is a great deal of latitude for practice variation and preference in the use of these drugs, and (2) VACHS PCPs are very familiar with the need to reduce dosage in the elderly, so stating this might just "clutter" the interface unnecessarily. The real lesson learned here is that there will always be room for judgment and iterative refinement in adjusting the content of the system's knowledge, based on user comments and domain expert judgment.

### Presentation

From the standpoint of presentation, we wanted the system to have one page with a very limited number of links so that the PCP could easily preserve context when using the system. The use of hover boxes was designed to help in that regard. We also wanted the presentation to be as clear, intuitive, and helpful as possible.

### Specific Comments on Presentation

Different clinicians raised a number of specific issues. It became clear that some features of the interface that seemed obvious to the developers were not immediately obvious to some of the clinicians using the system, for example: (1) the fact that the comorbidity checkboxes were initially set based on material from the chart (ICD9 codes), but could be changed by the PCP; or (2) that in the visual display of treatment options, the different treatment modalities were arranged in horizontal rows.

A clinician commented that there is quite a bit of material on this single screen. Another clinician felt that the use of color could enhance the readability of the static Web pages. Several clinicians were particularly positive about the ability to integrate use of the CDS with the CPRS interface, for example, the ease of access via the Tools menu, and the ability to copy and paste material from the CDS into the EHR.

These comments made it clear that it was important that a clinician should be shown the system, either in a clinical conference or by a colleague before using it, so he/she would be comfortable with its organization and understand the full range of its features.

## Discussion

### Current Status and Future Directions

Neuropath/CDS is currently operational on a pilot basis in the VACHS EHR, requiring a password since it is still under development. A logical future goal would be to make the approach available as adjunct to care with no password required. This would allow us to explore issues such as how best to promote its use, how frequently it is used, the types of patients it is used for, and the types of knowledge that tend to be accessed by PCPs (eg, which links and hover boxes are viewed).

We are also interested in extending this model of CDS, or components of the model, to other clinical domains. A number of clinicians suggested other potential domains for applying this model of CDS, including other types of pain, such as low back pain and headache, as well as many other diseases beyond pain. Some clinical domains may be sufficiently circumscribed that an approach very similar to that of Neuropath/CDS would work well. At the same time, it is clear that other domains may be much more complex and may require considerably more than a single screen to deal adequately with the clinical issues involved. For example, in the treatment of headache, there are at least five common types of headache, each with distinct approaches to pharmacologic management, and each with more first-line drugs than NP. In addition, one might like to provide some interactive assistance with diagnosis of headache. As a result, a CDS for a domain like headache might use features of Neuropath/CDS, but might well need to be considerably more complex.

### Conclusions

A major source of CDS within the VA EHR currently consists of clinical alerts and reminders, which can be very time-consuming and frustrating for the PCP to manage. In building Neuropath/CDS, we wanted to provide a tool that is perceived as helpful and not burdensome to the busy PCP. Ideally the clinician could spend less than a minute interacting with the system, be exposed to information useful in managing a specific patient, and then move rapidly on to his/her next task.

Neuropath/CDS has been built and deployed on a prototype basis in the context of the VACHS EHR. The work to date has allowed us to explore: (1) various design and implementation issues relating to the system itself and to the underlying approach to CDS; as well as (2) a framework for assessment, with a particular emphasis on issues relating to focus, scope, content, and presentation.

## Abbreviations

CDS: clinical decision support
CPRS: computerized patient record system
EHR: electronic health record
ICD9: International Classification of Diseases, 9th Revision
KB: knowledge base
NP: neuropathic pain
PCP: primary care provider
TCA: tricyclic antidepressant
VA: Veterans Administration
VACHS: VA Connecticut Healthcare System
